# Improved cookstoves enhance household air quality and respiratory health in rural Rwanda

**DOI:** 10.1038/s41598-025-09863-6

**Published:** 2025-07-18

**Authors:** Andrea Cuesta-Mosquera, Henning Kothe, Leizel Madueno, Allan Mubiru, Christine Muhongerva, Thomas Müller, Jan Rupp, Dominik van Pinxteren, Manuela van Pinxteren, Katherine Ogurtsova, Vanessa Soppa, Miriam Wiese-Posselt, Mira Pöhlker

**Affiliations:** 1 Atmospheric Microphysics Department, Leibniz Institute for Tropospheric Research (TROPOS), 04318 Leipzig, Germany; 2 Buana e.V, 22767 Hamburg, Germany; 3 Atmosfair gGmbH, 12059 Berlin, Germany; 4 Safer Rwanda, P.B 7301, Kigali, Rwanda; 5 Department of Infectious Diseases and Microbiology, University of Lübeck, 23562 Lübeck, Germany; 6 Atmospheric Chemistry Department, Leibniz Institute for Tropospheric Research (TROPOS), 04318 Leipzig, Germany; 7 Institute of Occupational, Social and Environmental Medicine, Centre for Health and Society, Medical Faculty, University Hospital Düsseldorf, Heinrich Heine University Düsseldorf, 04223 Düsseldorf, Germany; 8 Institute of Hygiene and Environmental Medicine, Charité-Universitätsmedizin Berlin, Corporate Member of Freie Universität Berlin and Humboldt-Universität zu Berlin, 12203 Berlin, Germany

**Keywords:** Spirometry, Black carbon, Household air pollution, Biomass burning, PAH, Atmospheric science, Respiratory signs and symptoms, Environmental impact

## Abstract

Household air pollution (HAP) from biomass combustion in traditional cooking methods poses significant health risks, particularly in rural communities of low- and middle-income countries. Improved cookstoves (ICS), designed to enhance combustion efficiency and reduce emissions, have been promoted as a transitional alternative towards cleaner cooking. However, evidence of their benefits remains mixed and context-specific. A randomized controlled trial was conducted to evaluate the impact of introducing the Save80 ICS on the respiratory health of adults in rural settlements in Rwanda. The study comprised two assessment rounds, and participants (*n* = 1001) were divided into two groups: one using traditional cooking methods and one using improved cookstoves. Baseline and follow-up data were collected through structured questionnaires and lung function tests. Furthermore, HAP was measured in a field campaign at households cooking with the ICS or traditional methods. The primary outcomes included respiratory symptoms, spirometry (FVC, FEV_1_, and PEF), and exposure to particulate matter (PM_0.3−2.5_) and its components (EC, OC, BC, BrC, and PAH). We found that households using the ICS spent, on average, 34% less time cooking and had 77% lower indoor PM_0.3−2.5_ levels. BC and BrC exposure decreased by 50% and 78%, respectively; OC and TC concentrations were 58% and 45% lower. PAH concentrations showed inconsistent patterns, with most species presenting non-statistically significant changes, constraining objective conclusions. Over the study period (3 years), ICS users reported lower prevalence of cough (-11%) and mucus production (-9%), and showed better forced vital capacity than users of traditional methods. A comparison of lung function decline over time showed that the ICS users had lower deterioration of FVC over three years. This study documents the effects and benefits of introducing ICS. While limitations such as the lack of baseline HAP data during the first health assessment and inconclusive PAH concentrations constrain interpretations and quantified causality, the results contribute to the evidence on the health and indoor air pollution impacts of ICS adoption in rural East African areas.

**Supplementary Information:**

The online version contains supplementary material available at 10.1038/s41598-025-09863-6.

## Introduction

Roughly 2.3 billion people globally lack access to clean cooking methods and rely on solid biofuels and kerosene as primary energy sources^1^. This practice is responsible for hazardous household air pollution (HAP). Repeated exposure to pollution derived from cooking emissions increases the risk of developing respiratory infections and non-communicable diseases^2–4^ and the likelihood of unfavorable pregnancy outcomes in women and newborns^5,6^. Furthermore, exposure to HAP was responsible for 3.2 million premature deaths annually in 2019^7^. In Sub-Saharan Africa, cooking with wood has been found to increase mortality in children and women, who are highly vulnerable due to greater exposure^8^. To mitigate HAP and its negative health effects, improved cookstoves (ICS) have been developed. Compared to traditional methods, ICS have enhanced combustion efficiencies, contributing to reducing fuel consumption and deforestation^9,10^ cooking time^11^ and greenhouse gas emissions^12^. Consequently, several low- and middle-income countries promote ICS as part of their programs for cleaner cooking, public health improvement, and climate change mitigation.

Despite the large body of research documenting the impacts of ICS on air pollution, the evidence remains mixed and sometimes contradictory. Several studies have reported positive health impacts from interventions with ICS^13–15^. However, numerous authors have also found contradictory outcomes regarding HAP and health^16–18^. These mixed results are attributed to factors such as inconsistent or incorrect cookstove use or reduced susceptibility in individuals who have been chronically exposed to HAP. Furthermore, the effectiveness of ICS interventions varies depending on regional characteristics, including cultural practices, socioeconomic context, accessibility to cleaner technologies, and lifestyles. Variables such as the type of fuel and cookstove, and the local environment, also play an essential role in the extent and composition of cooking emissions^19–22^. These complexities highlight the persistent gap in knowledge regarding localized HAP and health data from communities relying on solid biofuels and traditional cooking methods^23,24^. This gap is especially relevant in East Africa, where access to improved cookstoves and clean cooking technologies remains limited and varies depending on the country. For example, only 6% of Kenyan households have access to safe cooking methods^25^; 10% of the population in Uganda has access to modern cooking fuels^26^; and less than 3% of rural households in Tanzania have access to less polluting cooking fuels and methods^27^. In Rwanda, 8.3% of the population could access clean fuels and cooking technologies in 2022. Nevertheless, the figures are vastly different when splitting between urban and rural areas: less than 1% have access in rural sites compared to 34% in urban settlements^27,28^. Approximately 93% of rural Rwandan households use biomass for cooking, and 66% use three-stone fires^9,29^. The country faces significant challenges related to HAP, which is primarily caused by the prevalent use of biomass fuels in traditional cooking methods. In rural areas, where access to cleaner cooking technologies is limited, HAP is a major contributor to increased morbidity. In this context, introducing ICS can reduce exposure to harmful air pollutants and benefit respiratory health.

Studies in Rwanda have shown that HAP produced by cooking emissions is of major concern. In Kigali, Kabera et al.^30^ conducted an indoor air pollution study, measuring particulate matter with a diameter equal to or less than 2.5 microns (PM_2.5_) and carbon monoxide (CO) concentrations in households. The authors found 24-hour mean concentrations of PM_2.5_ and CO of 93 µg m^−3^ and 35 ppm, respectively. Both pollutants exceeded the maximum limits recommended by the World Health Organization (WHO)^3,31^. Further research in Rwanda has documented associations between biomass cooking and chronic bronchitis and chronic obstructive pulmonary disease (COPD) in women^32,33^. In West Rwanda, Kirby et al.^13^ found a 25% reduction in respiratory infections in kids after an intervention in a rural community using natural draft rocket-style stoves replacing traditional cooking setups.

**Fig. 1:**
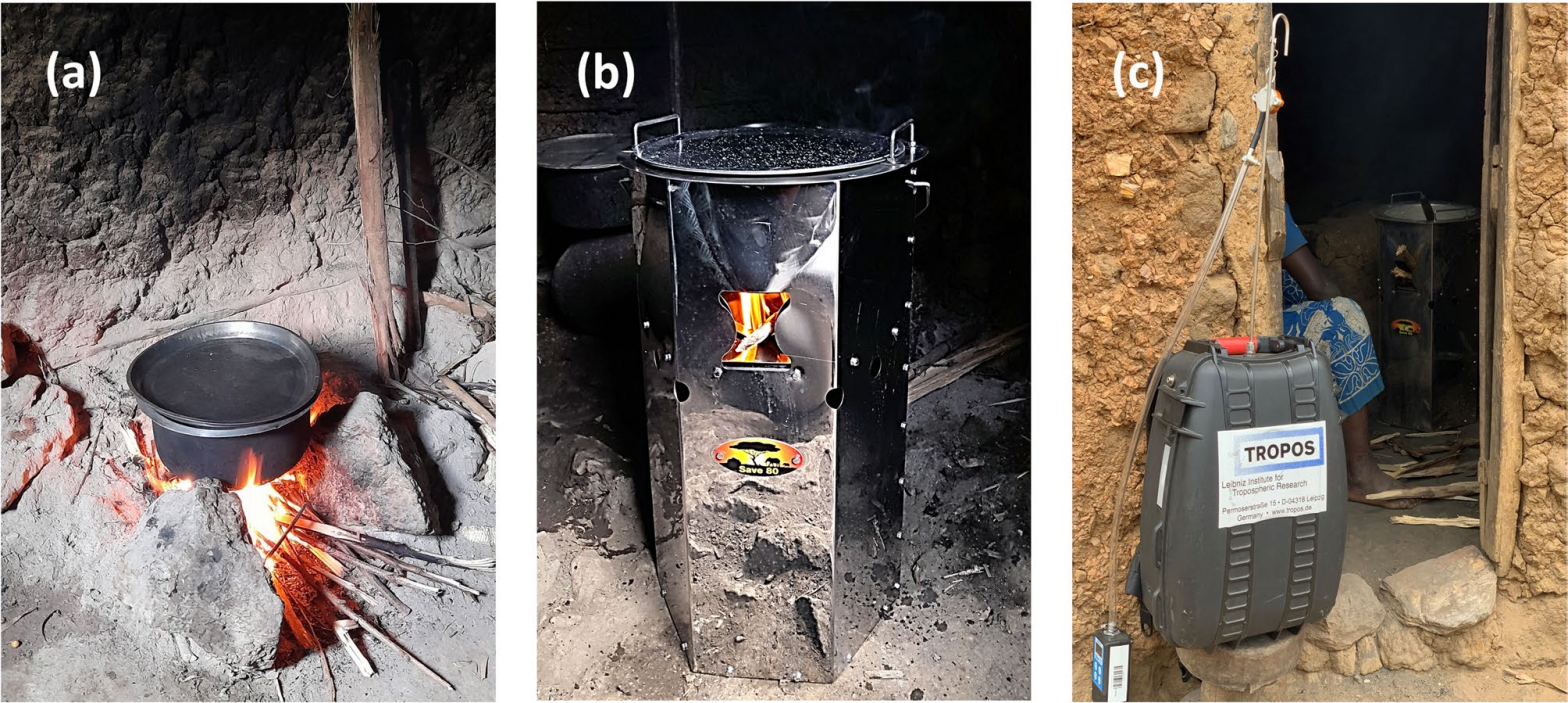
Photographs of (**a**) Traditional three-stone fire, (**b**) Improved cookstove type Save80, and (**c**) TROPOS-made portable backpack for online measurements and a low-volume portable sampler for PM filter collection.

Given the different types of stoves, fuels, and ways of use, there is still limited evidence on the effectiveness of interventions with ICS in the region. Among the various models introduced, the Save80 stove has seen relatively wide use. This is a wood-fueled, energy-efficient device with a natural draft configuration (Fig. 1). It offers significantly higher thermal efficiency than the traditional three-stone fires and other improved cookstoves, potentially reducing household wood consumption by up to 760 kg per year^34^.

Most field evaluations of HAP produced by cooking emissions have mainly focused on particulate matter, while limited investigation has been made into its components, such as black carbon (BC), brown carbon (BrC), and polycyclic aromatic hydrocarbons (PAHs). Exposure to BC and BrC impacts the respiratory and cardiovascular system; it has been linked to inflammation of the respiratory tract, deteriorated lung function, and aggravation of asthma and COPD symptoms^35,36^. PAHs, many of which are carcinogenic, contribute to oxidative stress, impaired pulmonary function, and increased risk of respiratory infections^37,38^. Localized and more comprehensive studies are essential for developing specialized interventions and policies to reduce health risks and improve life quality.

Previous research in Sub-Saharan Africa on health effects produced by introducing ICS shows the need for further investigation based on long-term follow-ups and more dedicated air pollution assessments^13,39^. Responses in respiratory health and other health-related parameters, such as reductions in respiratory and cardiovascular diseases, or adverse birth and child development outcomes, are not instantaneous and require longer observations^40,41^. The adherence of users, the prevalence of co-morbidities, and environmental variations can influence the outcomes from interventions and may not be observed in short-term studies.

This study aimed to evaluate the effects of introducing an improved cookstove (Save80) on the respiratory health of adults in rural communities in Rwanda. Furthermore, it was intended to assess the changes in household air pollution when transitioning from traditional cooking to the ICS. For this purpose, we conducted a randomized controlled trial (RCT) among 1001 individuals from two Rwandan villages. Participants were randomly allocated to an intervention group (*n* = 380) and a control group (*n* = 621). We assessed lung function (FEV_1_, FVC, FEV_1_/FVC, and PEF) and respiratory symptoms, including coughing, mucus production, and chest tightness, through a structured survey as part of a two-phase health assessment with three years in between. During the second phase of the study, we also characterized household indoor concentrations and exposure to PM, BC, BrC, organic carbon (OC), elemental carbon (EC), and PAHs using online instruments and collecting airborne particulate matter on filters. HAP measurements were performed in a subgroup of participants (*n* = 45), testing the pollution produced by traditional cooking and the ICS. Further details of the procedures are provided in the Methodology section. We hypothesize that using ICS instead of traditional methods for cooking results in better respiratory health outcomes for the study participants, as evidenced by improved respiratory symptoms and measured lung function parameters.

## Methods

### Study sites

The study was conducted in the districts of Musanze and Gatsibo in Rwanda (Fig. S1). Musanze district (1.3°S, 29.3°E, average altitude of 1860 m a.s.l., 476.500 inhabitants) is located in northwest Rwanda in the country’s most mountainous region, spanning an area of 530 km^2^^42^. The local climate is mainly humid, with an average temperature ranging between 13 and 18 °C, and maximum precipitation occurring in April and November (1300 to 1600 mm per year^43,44^). Gatsibo district (1.6°S, 30.4°E, 1465 m a.s.l., 551.100 inhabitants) is located in eastern Rwanda, covering an area of 1585 km^2^. The predominant geography consists of flat land and small elevations^45^. The climate is mainly hot and dry, with an average temperature of 25 to 27 °C and increased precipitation from March to May and October to December (700 to 1100 mm per year^44^. A significant fraction of the population in the study sites relied on agriculture as their primary income source: 57% and 72% of the employed adults in Musanze and Gatsibo worked as independent farmers, respectively^46,47^.

Firewood was the most common fuel used for cooking by Musanze (78%) and Gatsibo (92%) households^48,49^. Wood was collected from surrounding bushes, trees, and parcels, or bought from neighbors or nearby sellers. Traditional cooking primarily involved indoor open fires using a “three-stone” setup, where stones were arranged to support a cooking pot and confine the firewood (see Fig. 1a). Variations of the three-stone fire were also observed, such as the “u-shaped” setup, which constituted a fixed built-in structure used to contain firewood (see Fig. S2a).

### Study design

We conducted a randomized controlled trial in adults from the two study regions over a three-year period (September 2019 to May 2022), in which blinding was not possible due to the nature of the intervention. Participants were recruited through local farming cooperatives with the support of the NGO Safer Rwanda, focusing on adults responsible for cooking in their homes. Details about the study were provided to the communities in spoken and written form in French and Kinyarwanda. Following an initial expression of interest, participants were screened based on predefined inclusion and exclusion criteria (being at least 18 years old, not having chronic respiratory diseases, and not having an improved cookstove at home). Participation was entirely voluntary, without any financial or material compensation. Both oral and written consent were obtained from each participant. The ethics committee from the University of Lübeck, in Germany, assessed and approved the study protocol (protocol reference number 19–309), which follows the ethical principles of the World Medical Association Declaration of Helsinki from 2013, the most recent version of the Declaration by the time of the design and execution of the study.

The assignment of Save80 cookstoves to households was done through a simple random allocation process with a numbering generator. Participants were grouped into an intervention group and a control group. The intervention group received the stove at the beginning of the study, while the control group received it at the end. After receiving the stoves, participants were trained to use the devices. Safer Rwanda and local community leaders performed regular meetings, phone calls, and visits to households to verify the continuity and correct use of the stoves over the study period.

The first and second health assessments were conducted in 2019 and 2022, respectively (further details are provided in Sect. Health assessment).

Household air pollution measurements were performed only in 2022 during the second health assessment. The HAP measurements were performed on a subsample of households that did not receive the ICS during the first round, i.e., the control group, to build a baseline on air pollution before and after the intervention. The households monitored during the HAP assessment were selected considering their accessibility from the community halls serving as operational centers for the health assessments, since the batteries of mobile instruments need to be charged to operate prior to each measurement round (many houses lacked an electricity supply by the time of the study). HAP was measured over two rounds in 2022: in the first, households cooked using traditional methods, while in the second, households cooked using an ICS type Save80 after receiving training on correct stove usage. Further details on the HAP assessment are given in Sect. Exposure assessment.

#### The Save80 cookstove

The Save80^®^ is a natural draft stove built on stainless steel and fueled using firewood. It consists of a pot and a quadratic combustion chamber underneath, and small wood sticks are inserted into the combustion chamber through a frontal opening (see Fig. 1b). The cookstove was characterized under laboratory-controlled conditions at the Institute of Combustion Technology, RWTH, in Aachen, Germany, and showed an improvement factor of 4.7 in thermal efficiency analysis compared to the traditional three-stone method^15^. In addition, the participants received a polypropylene heat-retaining box (Wonderbox^®^, Fig. S2b), created to store and keep the food warm for a longer time, preventing them from having to warm up their food multiple times a day.

#### Health assessment

In the first round of examinations starting in 2019, we measured the participants’ lung function and administered a health questionnaire. The questionnaire was based on the RESPIRE study^16^ which integrated the COPD Assessment Test (CAT) and questions on cooking practices (time spent cooking per day, main cooking fuel, kitchen location -indoor/outdoor-, chimneys), and living conditions. Participants were asked about the occurrence and frequency of the following symptoms: cough, wheezing, chest tightness, and ocular itchiness. Furthermore, we asked whether they recently had or have other disease diagnosis (e.g., asthma, COPD, allergies, heart disease) and whether they were active or passive smokers. Three years later (in 2022), we carried out a second health assessment on the participants. The same health outcomes were measured in the second assessment, and the same questionnaire was applied.

Spirometry was performed following the guidelines from the European Respiratory Society and the American Thoracic Society (ERS/ATS^50^), measuring forced vital capacity (FVC), forced expiratory volume in 1 s (FEV_1_), and peak expiratory flow (PEF). Airway obstruction was defined as FEV_1_/FVC ratio < 70%. Repetitive measurements were carried out until at least three flow-volume curves were obtained that met the ERS/GOLD criteria^51,52^. We used a mobile spirometer Vyntus™ SPIRO PC-Spirometer (VYAIRE), in combination with a mouthpiece with an integrated bacterial filter and a nose clip. Air volume calibration of the spirometer was performed using a calibration pump at regular intervals throughout the day, including every morning, afternoon, and after significant weather changes. The meteorological parameters required for the calibration (temperature, humidity, and air pressure) were measured using a portable weather station (Technoline WS 6765).

The health assessments took place in community halls accessible to the study participants. The health questionnaire and the spirometry measurements were conducted and supervised by medical doctors who trained personnel from Safer Rwanda, supporting the health assessments.

#### Exposure assessment

In the second examination round, HAP was assessed in a subsample of households that had cooked exclusively using traditional methods in the last three years. The HAP measurement protocol consisted of three stages: (i) initial measurements 10 to 15 min before the cooking fire started, (ii) measurements during cooking with the fire on, and (iii) 10 to 15 min after the cooking when the fire had been turned off. An example of the time-resolved measurements is included in the Supplementary Information (Fig. S1).

BC, BrC, and PM mass concentrations were monitored at each household with high temporal resolution (< 1 min, Table 1). Additionally, we collected totalized PM_10_ filter samples during the cooking periods. Time-resolved BC was measured using a portable, small-sized light absorption photometer, the microAeth^®^ model MA200 (Aethlabs). The MA200 collects aerosol samples on a polytetrafluoroethylene (PTFE) filter tape, creating a 3-mm-diameter sample spot. The photometer continuously measures the light attenuation of the aerosol particles deposited on the filter tape at five wavelengths (375, 470, 528, 625, and 880 nm). The attenuation serves to estimate the aerosol mass concentrations of BC (and BrC). Further details on the instrument’s operating principles can be found in the literature^53,54^.

**Table 1:** Instrumentation used in HAP measurements.

Measurement	Instrument and manufacturer	Operation principle	Operating conditions	
			Time resolution	Configuration
Black carbon (eBC, µg m^−3^)	microAeth^®^ MA200, Aethlabs	Measurements of light attenuation of a filter loaded with aerosol particles, converted to BC mass using the optical absorbance per mass unit	10 s	Flow rate: 100 mL min^−1^ λ: 880 nm Detection limit: 30 ng m^3^ with 5 min time base, at 150 mL min^−1^ flow rate^55^
Particulate matter (PM, µg m^−3^)	Optical particle size spectrometer OPSS, model 3330, TSI Inc	PNSD based on scattering of light of aerosol particles	10 s	Size range: 0.3–10 μm Detection limits: 0 to 3000 particles cm^−3^
PM filters for laboratory analyses	Portable pump, model Gilian 12, Sensidyne	Collection of aerosol particles on a filter surface	1 Filter per household per cooking method	Flow rate: 10 L min^−1^ 37 mm quartz-fiber filters

PM concentrations were estimated using particle number size distributions (PNSD) measured with Optical Particle Size Spectrometers model 3330 (OPSS, TSI Inc). In brief, the instrument measures size- and time-resolved aerosol light scattering to determine the number of particles within an optical diameter range of 0.3 to 10 μm. The particles pass through a laser beam, producing a light pulse. The intensity of this pulse is then measured to determine the number and size of the particles in real time. The sizing calibration of the OPSS is performed in the laboratory using polystyrene latex (PSL) spheres with a specific refractive index. Further information on the instrument’s operation can be found elsewhere^56^. To calculate PM mass concentrations from the PNSD, we assumed spherical aerosol particles with a density of 1.4 g cm^−3^^57^. We also applied a refractive index correction to adjust the scattering-based measurements of the aerosols monitored indoors since their optical properties differ from those of PSL (used in calibration). We used a complex refractive index representative of biomass-burning aerosol particles^58^. The PM masses were estimated in two size ranges, 0.3–1 μm (PM_0.3−1_) and 1–2.5 μm (PM_1−2.5_). The coarse PM fraction (PM_2.5−10_) was not calculated, given the considerable uncertainty the refractive index correction added to the larger aerosol sizes^59^. Further details are given in the Supplementary Information.

The MA200s and OPSSs were carried inside TROPOS-made portable backpacks (Fig. 1c) to protect the instruments and facilitate their transport^59,60^. The backpacks were constructed with waterproof hard cases and had 1-m stainless-steel inlets to capture the aerosol sample. Inside the system, aerosols first passed through a silica gel dryer to control changes in humidity before reaching the MA200 and OPSS, which are connected to a microcomputer for data storage. During the measurements, the backpacks were located indoors, 2–3 m from the cooking fire or stove.

Instrument quality assurance and quality control procedures followed the recommendations of the World Calibration Centre for Aerosol Physics (WCCAP) in Leipzig (more details are given in the Supplementary Information).

PM_10_ filter samples were collected at a subgroup of households included in the HAP measurements using a low-volume portable sampler model Gilian 12 (Sensidyne, Fig. 1c). Before the campaign, the filters were preheated for 24 h at 105 °C to reduce blank values and stored frozen after measurements. In the laboratory, we determined mass concentrations of organic and elemental carbon (OC and EC), total carbon (TC), and particulate polycyclic aromatic hydrocarbons (PAH). Twenty-two PAH species were analyzed (PAH_22_), 13 of which are listed on the group of sensitive PAHs prioritized by the U.S. EPA (PAH_13_, underlined): *Fluorene*, *Phenanthrene*, *Anthracene*, *Fluoranthene*, *Pyrene*, Retene, Benzo(b)naphtho(1,2-d)thiophene, Cyclopenta(cd)pyrene, *Benzo(a)anthracene*, *Chrysene(+ Triphenylene)*, 2,2-Binaphthyl, *Benzo(b)fluoranthene*, *Benzo(k)fluoranthene*, Benz(e)pyrene, *Benz(a)pyrene*, *Indeno(1*,*2*,*3-cd)pyrene*, *Dibenzo(ah)anthracene*, *Benzo(ghi)perylene*, Coronene, 9 H-Fluorenone, 9,10-Anthracenedione, and 1,2-Benzanthraquinone.

The OC/EC mass concentrations were determined using a thermal-optical method, following the EUSAAR-2 Protocol^61^ with a Sunset Laboratory dual carbonaceous analyzer. PAHs were determined from two circular pieces of filter (6 mm diameter, 56.5 mm^2^) using a Curie-point pyrolyzer (JPS-350, JAI) coupled to a GC-MS system (6890 N GC, 5973 inert MSD, Agilent Technologies). More details about the analytical methods can be found in Mateus-Fontecha et al.^62^ and Neusüss et al.^63^.

We calculated the total HAP exposure ($\:{\varepsilon}_{i}$) applying the following equation to the online measurements data of BC, BrC, PM_0.3−1_, and PM_0.3−2.5_:

1 $${\varepsilon}_{i}={\int}_{{t}_{1}}^{{t}_{2}}{c}_{i}\left(t\right)dt$$

where *c*_*i*_ is the measured concentration of pollutant *i*, and *t*_1_ and *t*_2_ are the starting and ending cooking times.

### Data analysis

#### Health data

**Power analysis**.

A power analysis was conducted prior to participant recruitment to determine the minimum sample size required for the study. The analysis aimed to ensure that the study would have sufficient power (set at 80%) to detect a meaningful difference between the intervention group (ICS users) and the control group (traditional cooking). Based on previous studies^14^ a small effect size was anticipated (Cohen’s d = 0.2), and the significance level (alpha) was set at 0.05. As a result, it was estimated that a minimum of 150 participants would be needed per group (control and intervention), resulting in a total sample size of 300 participants. To account for potential dropouts, the sample size was adjusted by 40%, considering dropout rates from other studies^15^ bringing the final recruitment target to 420 participants per village, thus 840 in total. The power analysis was done in the software G*Power version 3.1.9.4^64^. Given the conservative nature of the initial power assumptions and the study capabilities, 1000 participants were reached between both villages to ensure robustness in detecting the hypothesized effects.

**Respiratory symptoms and lung function analysis**.

In the cross-sectional analysis, the differences in reported respiratory symptoms (categorical variables) between the two participant groups were examined using a Chi-square test. Differences in lung function (continuous variables) were assessed using non-parametric Wilcoxon rank sum tests following a normality check with the Shapiro-Wilk test.

In the longitudinal analysis, we calculated the temporal changes by comparing the first assessment with the follow-up within each group of participants. Statistically significant differences were tested using the Wilcoxon test for paired samples. The significance level for the statistical tests was always set to α = 0.05.

**Exposure data**.

Differences in HAP measurements between the two groups were assessed using non-parametric Wilcoxon signed-rank tests, following Shapiro-Wilk tests for normality. The significance level for the statistical tests was always set to α = 0.05.

The data processing and analyses were conducted using the software R version 4.4.1^65^.

## Results

### Participants characteristics

**Fig. 2:**
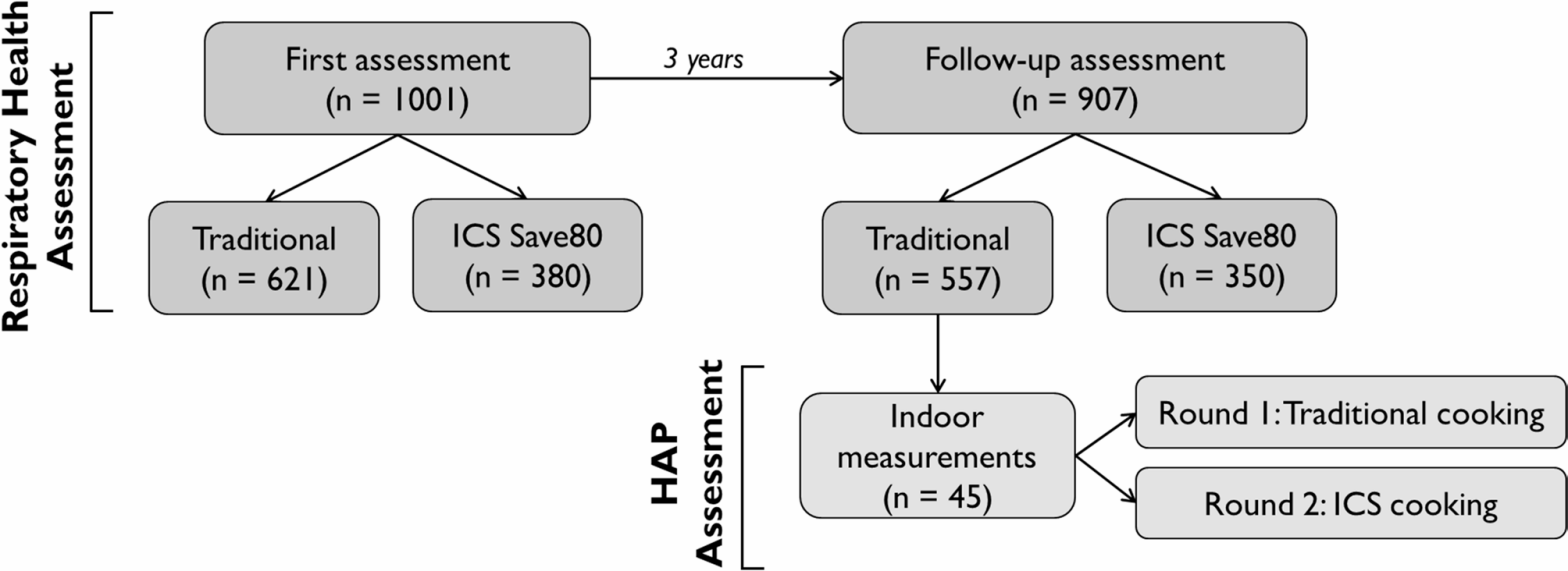
Flowchart of the study participants of the health examination and the HAP measurements.

Initially, 1001 individuals (499 from Musanze and 502 from Gatsibo) were included in the study. The characteristics of the participants are shown in Table 2. At baseline, 380 participants had received an ICS and used it over three years (2019 to 2022), while the remaining 621 participants continued to cook with traditional methods during the same period (Fig. 2). Most participants were women (86%, *n* = 862), with an average age of 42.2 ± 12.2 years (range 17–92 years). Male participants (14%, *n* = 139) had an average age of 42.5 ± 12.8 years (range 18–86 years). Most participants were nonsmokers (97%), and 12% reported being exposed to secondhand cigarette smoke. The mean accumulated years of cooking in the whole group was 20 ± 12.6 years. Traditional cooks reported average cooking years of 19.6 ± 11.7, while Save80 cooks reported 25 ± 12.5 years. Wood was the primary cooking fuel (95%), followed by charcoal (5%) and other fuels (including bush, leaves, and liquified petroleum gas [LPG], 0.3%) in the whole group.

**Table 2:** Demographics of the study participants at the baseline.

Variable	Total (*n* = 1001)	Traditional (*n* = 621)	Improved stove Save80 (*n* = 380)
Age (years; mean ± SD)	42.5 ± 12.5	42.1 ± 12.3	42.2 ± 13.2
**Gender**			
Female [n (%)]	862 (86%)	540 (87%)	322 (85%)
Male [n (%)]	139 (14%)	81 (13%)	58 (15%)
Height (cm; mean ± SD)	159 ± 7	159.8 ± 7	159.8 ± 7
Weight (kg; mean ± SD)	61.23 ± 9.9	59.9 ± 10.3	60.9 ± 10.8
**Occupation**			
Farmer [n (%)]	929 (93%)	577 (93%)	352 (93%)
Other [merchant, teacher, housewife, etc.; n (%)]	72 (7%)	44 (7%)	28 (7%)
**Smoking**			
Yes [n (%)]	34 (3%)	25 (4%)	9 (2%)
No [n (%)]	967 (97%)	596 (96%)	371 (98%)
**Passive smoker**			
Yes [n (%)]	116 (12%)	75 (12%)	41 (11%)
No [n (%)]	885 (88%)	546 (88%)	339 (89%)
**Cooking**			
Years as a primary cook (mean ± SD)	20 ± 12.6	19.6 ± 11.7	25 ± 12.5
**Cooking fuel**			
Wood [n (%)]	949 (94.8%)	590 (95%)	359 (94.5%)
Charcoal [n (%)]	49 (4.9%)	29 (4.7%)	20 (5.2%)
Other (e.g., bush and leaves, LPG; [n (%)])	3 (0.3%)	2 (0.3%)	1 (0.3%)

In the second round of health examinations in 2022, questionnaire data were obtained from 907 participants (495 from Musanze and 412 from Gatsibo); this represents a sample decrease of 94 persons (9.4%) in the follow-up assessment. The main reasons were participant relocation or loss of contact. The participants who dropped out of the study were not statistically different from those who remained (age, *P-value* = 0.231; gender, *P-value* = 0.117; smoking history, *P-value* = 0.452; and medical conditions, *P-value* = 0.288). In the remaining fraction, 557 participants cooked using traditional methods, and 350 used the ICS (Fig. 2). No significant differences were observed between the remaining participants and those who did not complete the study.

### Health outcomes

During the first assessment, 1001 individuals responded to the questionnaire, and acceptable spirometry data were obtained from 935 participants. At the baseline, no significant differences in symptoms and lung function were observed between the intervention and control groups. Obstructive pulmonary disease at baseline was found in 32 participants (3%, FEV_1_/FVC < 70%).

**Fig. 3:**
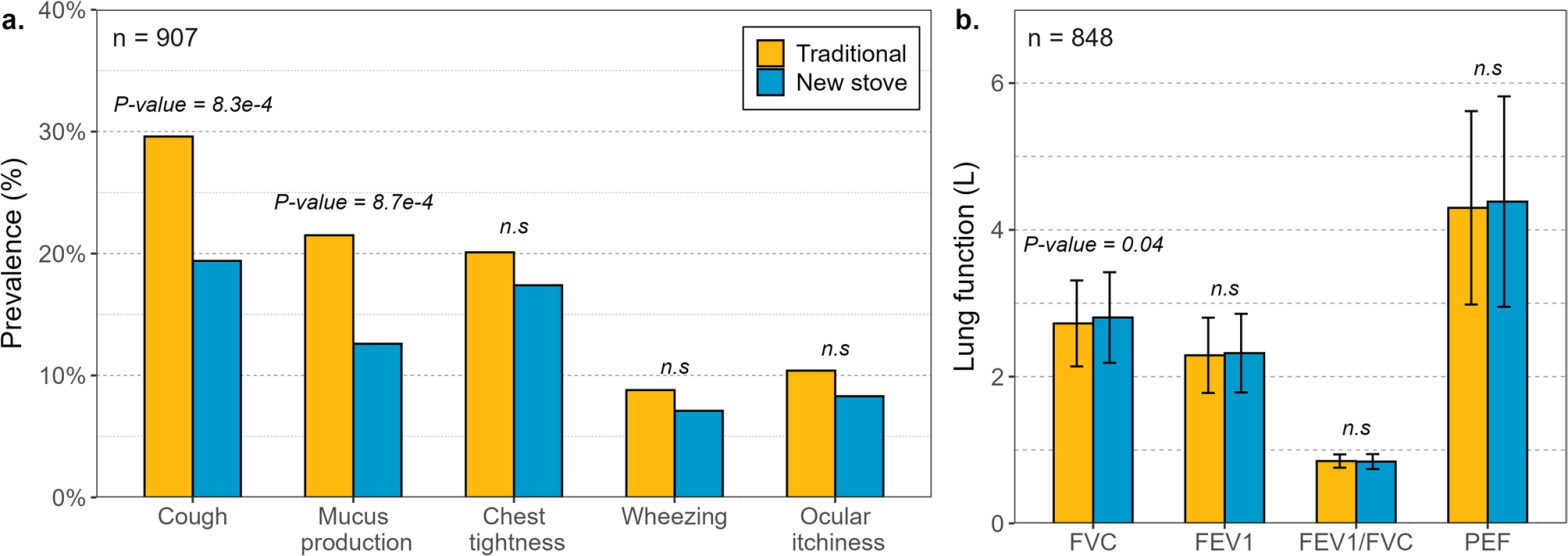
Reported health parameters for participants using traditional cooking and improved cookstoves at the follow-up assessment. (a) Percentage of prevalence of clinical symptoms and (b) Median values of lung function variables with error bars representing the 95% CI of the median.

In the follow-up, 907 participants responded to the questionnaire, and acceptable lung function data were obtained from 848 individuals (the sample of spirometry observation data decreased by 9.3%). In this phase, those participants who used the improved stoves over three years reported a lower prevalence of symptoms than participants who remained with traditional cooking (Fig. 3a; Table 3), with significant reductions for cardinal symptoms like coughing (29% traditional cooks vs. 19% ICS users, Chi-square test, *P-value* < 0.001) and mucus production (13% traditional cooks vs. 22% ICS users, Chi-square test, *P-value* < 0.001). Only slight, but not significant, improvements were found for nonspecific symptoms: chest tightness (*P-value* = 0.446), wheezing (*P-value* = 0.361), and ocular itchiness (*P-value* = 0.346).

**Table 3:** Descriptive statistics for household air pollution and lung function.

Variable	Traditional cooking (mean ± SD)	Improved stove (mean ± SD)	*P*-value
**Exposure** (*n* = 45)			
Cooking time (min)	46.6 ± 13.2	30.8 ± 7.6	6.2e-08*
BC (µg m^−3^ hour^−1^)	60.1 ± 49	29.9 ± 19.8	1.9e-04*
BrC (µg m^−3^ hour^−1^)	163.1 ± 120	35.2 ± 22.9	7.4e-10*
PM_0.3−1_ (µg m^−3^)	20.4 ± 21.1	5.9 ± 6.5	2.9e-08*
PM_0.3−2.5_ (µg m^−3^ hour^−1^)	167.1 ± 204.1	37.6 ± 41.2	6.2e-08*
**Concentrations** (*n* = 21)			
EC (µg m^−3^)	37.9 ± 22.8	62.2 ± 39.7	5.6e-02
OC (µg m^−3^)	329.8 ± 260.7	138.6 ± 79.4	3.5e-03*
TC (µg m^−3^)	367.7 ± 277.4	200.8 ± 105.2	4.2e-02*
EC/OC	0.14 ± 0.09	0.48 ± 0.27	5.6e-07*
PAH_13_ (µg m^−3^)	1.0 ± 0.9	1.9 ± 1.8	6.7e-02
PAH_22_ (µg m^−3^)	4.8 ± 3.6	3.8 ± 3.5	7.6e-01
**Respiratory symptoms** (*n* = 907) ^**a**^	*n* = 557	*n* = 350	
Coughing (%)	18.2	7.5	8.3e-04*
Mucus (%)	13.2	4.8	8.7e-04*
Chest tightness (%)	12.3	6.7	4.5e-01
Wheezing (%)	5.4	2.7	3.6e-01
Ocular itchiness (%)	6.4	3.2	3.5e-01
**Lung function** (*n* = 848)^a^	*n* = 520	*n* = 328	
FVC (L)	2.74 ± 0.58	2.82 ± 0.62	4.4e-02*
FEV_1_ (L)	2.30 ± 0.51	2.35 ± 0.54	1.8e-01
FEV_1_/FVC (%)	0.84 ± 0.1	0.84 ± 0.1	2.8e-01
PEF (L)	4.46 ± 1.3	4.46 ± 1.4	9.4e-01

^a^Health data correspond to the follow-up assessment.

*P-value < 0.05, significant difference.

After three years, the lung function of the ICS users showed a higher forced vital capacity (FVC) than that of traditional cooks (Fig. 3b, median = 2.82 L for ICS users vs. 2.74 L for traditional cooks, Wilcoxon test, *P-value* = 0.044). However, no statistically significant differences were found for FEV_1_ (median = 2.30 L for ICS users vs. 2.35 L for traditional cooks, *P-value* = 0.179) and PEF (median = 4.46 L for ICS users vs. 4.46 L for traditional cooks, *P-value* = 0.936). The FEV_1_/FVC ratio in the ICS users’ group (median = 84%) was not statistically significantly different from that of traditional cooks (median = 85%, *P-value* = 0.288). Obstructive pulmonary disease was found in 50 participants (6%, FEV_1_/FVC < 70%). From these, 26 individuals were traditional cooks (mean FEV_1_/FVC = 62%), 24 individuals were ICS users (mean FEV_1_/FVC = 60%), and no significant difference was found for individuals with FEV_1_/FVC < 70% when comparing both groups.

### Temporal evolution of respiratory health

Considering the differences observed in the cross-sectional assessment, we analyzed the change in FVC for both groups of participants between the first (2019) and follow-up (2022) assessments. A decrease was observed in the whole sample after three years, which is expected as lung function naturally declines over time. However, this decline was relatively lower within the intervention group (mean ∆FVC = −0.02) compared to the control group (mean ∆FVC = −0.16). The differences in ∆FVC between both groups of participants were statistically significantly different (∆FVC_Save80_ vs. ∆FVC_Traditional_, *P*-value < 0.001), favoring the intervention group.

The temporal variation in self-reported symptoms revealed a decrease in both groups with respect to the baseline, yet a larger change was seen in the intervention. Despite this observation, the temporal reduction was only significant for the symptoms of coughing (20% reduction, Wilcoxon, *P-value* = 0.003) and chest tightness (15% reduction, Wilcoxon, *P-value* = 0.009).

### Household air pollution

**Fig. 4:**
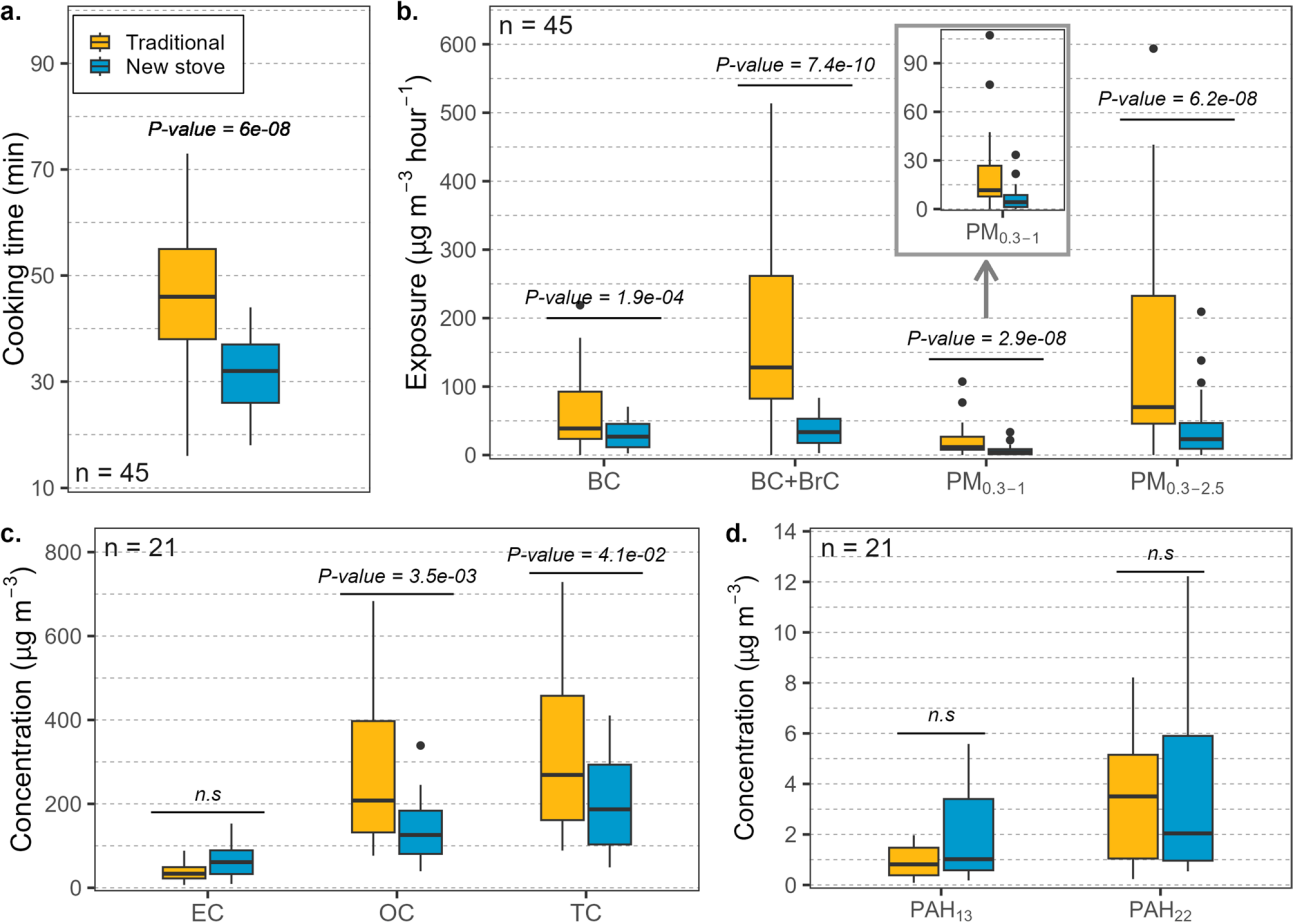
Comparison of (**a**) cooking time, (b) pollutants exposure rate, (**c**) carbonaceous pollutant exposure concentrations, and (**d**) PAH mass concentrations measured for each cooking method. The lower and upper borders of the boxes represent the first and third quartiles in which the middle 50% of the statistical variables are located, the horizontal black lines inside the boxes represent the median, and the whiskers represent the minimum and maximum values without outliers. The black dots represent the outliers.

Using the ICS Save80 reduced the average cooking time by 34% (Fig. 4a; Table 3), resulting in lower exposure to household air pollution (Fig. 4b; Table 3). This decline was estimated from the cooking times monitored during the HAP measurements and corroborated by the responses provided by participants in the questionnaire during the second health assessment (mean cooking time, traditional = 3 h, ICS users = 2 h, *P-value* < 0.001). The mean exposure to BC and BrC(+ BC) decreased by 50% (Wilcoxon test, *P-value* < 0.01) and 78% (*P-value* < 0.001), respectively. During traditional cooking, BC exposure was 60 ± 49 µg m^−3^ h^−1^ and decreased to 30 ± 20 µg m^−3^ h^−1^ during the improved stove use. Regarding the organic-light-absorbing carbon (BrC), the mean hourly exposure was 163 ± 120 and 35 ± 23 µg m^−3^ h^−1^ for traditional cooking and the ICS, respectively.

The carbonaceous aerosols collected in the quartz filters also significantly reduced the organic fraction (Fig. 4c). The OC concentration decreased by 58% (Wilcoxon test, *P-value* = 0.004), passing from 330 ± 261 µg m^−3^ hour^−1^ to 139 ± 79 µg m^−3^ hour^−1^, on average. However, the EC concentration showed increments for the ICS, passing from 38 ± 23 µg m^−3^ hour^−1^ to 62 ± 40 µg m^−3^ hour^−1^ (+ 63%), although the difference was not statistically significant (*P-value* = 0.05). Despite the apparent increment in EC, the average TC concentration decreased by 45% (*P-value* = 0.041), passing from 368 ± 277.4 µg m^−3^ hour^−1^ to 201 ± 105.2 µg m^−3^ hour^−1^. The mean EC/OC for traditional cooking was 0.14 (P.10 = 0.08, P.90 = 0.26), while EC/OC = 0.47 for the ICS (P.10 = 0.21, P.90 = 0.77).

PM concentrations were obtained from the particle number size distributions measured between 0.3 and 2.5 μm. The exposure to the finest fraction (0.3 to 1 μm, PM_0.3−1_) was comparatively lower than that of the larger fraction (0.3 to 2.5 μm, PM_0.3−2.5_) for both cooking methods. The mean PM_0.3−1_ exposure during traditional cooking was 20 ± 21 µg m^−3^ hour^−1^, significantly higher than the PM_0.3−1_ exposure for Save80 use, equivalent to 6 ± 4 µg m^−3^ hour^−1^ (Wilcoxon test, *P-value* < 0.001). In terms of PM_0.3−2.5_, the mean exposure during traditional cooking was 167 ± 207 µg m^−3^ hour^−1^, significantly higher than the mean exposure of 38 ± 41 µg m^−3^ hour^−1^ measured during the improved stove use (*P-value* < 0.001).

The total concentration of PAHs showed no statistically significant changes after the introduction of the ICS. The concentrations of the United States Environmental Protection Agency (U.S. EPA)-sensitive PAHs analyzed (PAH_13_) showed apparent increments with the ICS use by ~ 1 µg m^−3^ (Fig. 4d; Table 3); however, the difference with the traditional cooking measurements was not statistically significant (*P-value* = 0.07). The mean concentration of all species (PAH_22_) decreased by 20% during the improved cookstove use; however, the difference between the two cooking methods was not statistically significant (*P-value* = 0.08). In terms of individual PAH species (Table 4), we found increments and reductions in concentrations, with significant variations only for 9,10-Anthracedione (reduction, *P-value* = 0.001), 9 H-Fluorenone (increment, *P-value* = 0.0001), Anthracene (increment, *P-value* = 0.002), Benzo(gui)perylene (increment, *P-value* = 0.017), Benzo(k)fluoranthene (increment, *P-value* = 0.049), Coronene (increment, *P-value* = 0.021), and Phenanthrene (increment, *P-value* = 0.008).

**Table 4:** Comparison of each PAH species mass concentrations measured for each cooking method from 21 households at the follow-up.

PAH	Traditional cooking (µg m^−3^)		Improved stove (µg m^−3^)		*P*-value
	Mean ± SD	Q_0.25_ – Q_0.75_	Mean ± SD	Q_0.25_ – Q_0.75_	
1,2-Benzanthraquinone	0.15 ± 0.12	0.04–0.20	0.08 ± 0.09	0.00-0.10	1.84e-01
2,2-Binaphthyl	0.01 ± 0.01	0.00-0.01	0.01 ± 0.02	0.00-0.02	2.66e-01
9,10-Anthracenedione	1.67 ± 1.81	0.34–2.23	0.36 ± 0.57	0.08–0.37	1.22e-03*
9 H-Fluorenone	0.05 ± 0.04	0.04–0.06	0.14 ± 0.09	0.09–0.18	1.22e-04*
Anthracene	0.00 ± 0.00	0.00–0.00	0.01 ± 0.01	0.00-0.02	2.25e-02*
Benzo(a)anthracene	0.14 ± 0.11	0.05–0.19	0.22 ± 0.21	0.07–0.34	1.53e-01
Benz(a)pyrene	0.23 ± 0.18	0.09–0.33	0.43 ± 0.39	0.12–0.74	1.19e-01
Benz(e)pyrene	0.08 ± 0.05	0.03–0.11	0.14 ± 0.11	0.05–0.23	5.8e-02
Benzo(b)fluoranthene	0.21 ± 0.15	0.09–0.31	0.38 ± 0.32	0.13–0.64	6.73e-02
Benzo(b)naphtho(1,2-d)thiophene	ND	ND	ND	ND	ND
Benzo(ghi)perylene	0.08 ± 0.06	0.03–0.12	0.20 ± 0.14	0.08–0.35	1.66e-02*
Benzo(k)fluoranthene	0.07 ± 0.05	0.03–0.10	0.15 ± 0.13	0.05–0.25	4.94e-02*
Chrysene(+ Triphenylene)	0.04 ± 0.03	0.02–0.06	0.06 ± 0.06	0.02–0.10	1.73e-01
Coronene	0.01 ± 0.02	0.00-0.03	0.03 ± 0.04	0.00-0.08	2.09e-02*
Cyclopenta(cd)pyrene	0.54 ± 0.58	0.12–0.85	0.90 ± 0.97	0.18–1.54	2.17e-01
Dibenzo(ah)anthracene	0.01 ± 0.01	0.00-0.02	0.02 ± 0.03	0.00-0.05	5.87e-02
Fluoranthene	0.05 ± 0.09	0.00-0.06	0.09 ± 0.12	0.00-0.15	1.68e-01
Fluorene	ND	ND	ND	ND	ND
Indeno(1,2,3-cd)pyrene	0.13 ± 0.10	0.05–0.19	0.27 ± 0.22	0.08–0.47	6.7e-02
Phenanthrene	0.02 ± 0.02	0.00-0.03	0.04 ± 0.03	0.03–0.06	7.92e-03*
Pyrene	0.06 ± 0.10	0.00-0.08	0.11 ± 0.15	0.00-0.19	1.42e-01
Retene	ND	ND	ND	ND	ND

ND: Not detected.

*P-value < 0.05, significant difference.

## Discussion

Biomass-fueled improved cookstoves have become a common strategy to mitigate HAP produced by inefficient cooking in low- and middle-income countries, particularly in rural areas with limited infrastructure to obtain energy from cleaner sources. Nevertheless, understanding the effects of using the ICS on human health and household air pollution still requires further investigation, as these vary for each community depending on several factors such as the type of stove and fuel, cultural practices and behaviors, the physical environment, living conditions, and baseline health parameters. We investigated the impact of introducing an ICS type Save80 in rural Rwandan settlements through health assessments in adults and in-situ characterization of aerosol particle emissions. We compared and observed the temporal variation of the respiratory health of participants using traditional methods and the ICS. Furthermore, we measured the exposure and composition of aerosol particles produced by both cooking methods under real conditions. Our findings demonstrate that participants using ICS experienced reduced pollutant exposure, reported a lower prevalence of certain respiratory symptoms, and had higher lung function.

The cross-sectional comparison of groups after three years of adopting the ICS showed that the intervention group reported significantly less coughing (−11%) and mucus production (−9%) and had better forced vital capacity (FVC, 100 mL) compared to the control group. Slight differences in FEV_1_, PEF, and FEV_1_/FVC ratio were observed but were not statistically significant. These results may be associated with the limitations of spirometry measurements attributed to participant effort and cooperation dependency. In a longitudinal analysis, we compared changes in respiratory health within each group of participants from the first assessment to the follow-up. We found a decline in average lung function over the three years of the study in both groups. However, these declines or negative deltas in lung function (∆FVC) were comparatively lower in the group of participants using the ICS. The deterioration in lung function with aging has already been documented in multiple studies^66^. For instance, Musafiri et al.^67^ observed a negative correlation between FEV_1_ and FVC with age in healthy Rwandans; the authors developed mathematical correlations of lung function with age and height. Applying their parametrizations for a constant height (165 cm) and an average age of 42 years, we estimate a deterioration of 57 mL of air in terms of FVC over three years for Rwandan males and females. This declination was found to be lower than the one observed in our study for participants using traditional methods (∆FVC = −160 mL) but larger than average deltas calculated by ICS users (∆FVC = −20 mL).

Using the ICS contributed to a 34% reduction in average cooking time, which positively impacted HAP exposure. BC and BrC exposure decreased by 50% and 78%, respectively, and PM_0.3−1_ and PM_0.3−2.5_ exposure decreased by 70% and 77%. In addition, OC filter concentrations decreased by 58%; nevertheless, the average EC concentration increased by 63%, although the changes in EC were not statistically significant. This might be explained by the small sample size used for filter analyses (*n* = 21), suggesting that a larger sample is needed to draw stronger conclusions. Changes in EC led to differentiated EC/OC ratios for each cooking method (Traditional = 0.14, ICS = 0.48). Higher EC concentrations and EC/OC ratios for ICS have also been reported in other studies^21,68^ and are associated with the burning conditions and higher temperatures reached with the stoves, which disfavor the formation of organic material and favor complete combustion. Despite the apparent increment in EC, the TC concentrations measured during the ICS use were 45% lower than those from traditional cooking. In terms of overall HAP, the findings of the present study align with the results observed in similar research. In Nigeria, Onyeneke et al.^11^ assessed the effect of an intervention with the ICS type Save80 in a rural community; the authors found a reduction in cooking time of 38% among participants using the stove with respect to a control group using open fires. Furthermore, they observed reductions in fuel collection time, indoor carbon monoxide concentrations, and prevalence of sore eyes. Many other studies have also reported reductions in emissions and concentrations of air pollutants from the adoption of ICS^24,69,70^.

PAH concentrations showed no significant differences when comparing both cooking methods. Regarding PAH_13_, classified as potentially carcinogenic and mutagenic by the U.S. EPA, the mean concentration during the ICS was almost twice that measured during traditional cooking; however, these concentrations proved to be not significantly different and can be partially attributed to the limited sample size (*n* = 21) for PAH measurements. A subsequent power analysis was performed considering a small effect size, d = 0.29, calculated using the mean and standard deviation of the PAH concentrations measured before and after the ICS intervention, and alpha = 0.05. Results indicated that the statistical power to detect the observed difference was only 0.23, well below the conventional threshold of 0.80. This suggests a high probability of a Type II error (failing to detect a true effect due to insufficient sample size) and calls for caution when interpreting these non-significant findings. The observed variability in PAH levels may also arise from differences in combustion conditions inherent to stove design, particularly temperature profiles and oxygen availability during cooking. PAH formation is highly sensitive to these parameters: low- and middle-molecular-weight PAH tend to be emitted when biomass burning temperatures reach 400 °C, while high-molecular-weight PAH start forming at 500 °C, particularly under oxygen-deficient conditions^71^. At elevated combustion temperatures, Eriksson et al.^72^ reported increased yields in PAH formation during rapid combustion in hot, oxygen-deficient conditions, especially for high-molecular-weight species associated with carcinogenicity. The small combustion chamber of the Save80 stove likely promotes rapid heat buildup and elevated peak temperatures due to better insulation and reduced convective losses. However, the more enclosed combustion area may also promote oxygen-deficient microenvironments, particularly during ignition in high-flame phases, where pyrolytic paths dominate. These conditions can favor the formation of certain high-molecular-weight PAH, even if the total PM mass is reduced. A more detailed chemical analysis of the combustion phases and emissions from the Save80 stove could help determine whether such mechanisms are responsible for the PAH and EC profiles observed in this study.

Background (pre-cooking) online concentrations of BrC were evaluated as a proxy to infer whether particulate PAH concentrations were larger during the second round of HAP measurements and affected the concentrations determined during ICS use. No significant differences were found in the median BrC background concentrations between the measurements during each cooking method (BrC_background, traditional_ = 0.68, BrC_background, ICS_ = 0.77, *P-value* = 0.43, Fig. S4). These results suggest that observed differences in PAH concentrations are more likely attributable to variations in cooking or indoor emissions rather than changes in ambient or external sources. In Rwanda, Kalisa et al.^73^ characterized ambient PAH concentrations and found cooking emissions to be a significant fraction of PAH ambient concentrations in rural areas. In their study, PAH levels measured in rural settings were higher than those in urban background and lower than in urban roadside sites, except for Fluorene, Naphthalene, and Acenaphthene, which were highest in the rural areas compared to urban roadside and background sites. To clarify the health effects of PAHs caused by ICS use, further studies are needed, considering larger samples, longer-term assessments, and analyses of the inhalation and excretion mechanisms of PAH.

### Limitations

Several limitations of this study should be discussed. First, HAP measurements were performed only during the second phase of the health study and limited to a subset of participants. While this condition restricts the ability to quantify causal correlations between air pollution and health outcomes, the main goal of the study was to document the responses on HAP levels and respiratory health indicators, followed by the introduction of the stoves. Nevertheless, baseline HAP data are well represented by the pre-intervention measurements performed at households before the acquisition of the stoves. This is a valid approach, given that changes in HAP are observed instantaneously after alterations in the emission sources, while changes in health parameters may require larger periods. The evaluation of HAP was representative of the actual improvements that could have been experienced if measurements had been performed at the beginning of the study in 2019. Given the stable use of three-stone fires in this population over time, it is reasonable to assume that pre-intervention HAP exposure in the control group during the second phase reflects baseline conditions of HAP, and no significant changes occurred. Performing the HAP campaign during the second phase allowed us to recruit a larger and more representative subsample of participants (*n* = 45), providing sufficient statistical power for exposure assessment and comparison in terms of PM, BC, and BrC. Following this methodology, we detected significant differences in exposure to aerosol emissions between the two cooking methods, with improvements in most parameters measured after introducing the stoves. However, the results indicate that using ICS still poses a risk to human health, despite the improvements in exposure, given that pollution levels remained above the maximum limits recommended by the WHO^74,75^.

Other pollutants, such as ultrafine particles (UFP) and gaseous species, were not monitored. Improved cookstoves can produce considerable emissions of UFP, which may yield lung inflammation and contribute to the circulation of inflammatory mediators to the rest of the body^76^. Gaseous pollutants such as carbon monoxide (CO), carbon dioxide (CO_2_), and nitrogen oxides (NO_X_) may negatively impact the respiratory and cardiovascular systems. Chronic exposure to CO and CO_2_ has been associated with cardiovascular events and increased vulnerability of the heart to physiological stress^77^ as well as impairing cognitive performance function^78,79^. Exposure to high levels of NO_X_ generates detrimental effects on FEV_1_ and FVC^80^ and exacerbates the risk of developing asthma, COPD, and cardiac failure^81^. The absence of gaseous pollutant measurements constitutes a limitation in accurately characterizing the emissions associated with traditional methods and improved cookstoves. Besides being indicators for potential health risks, gases produced during wood and biomass burning are key indicators of combustion efficiency, temperature, and fuel composition. Including them in further studies is essential for a complete overview of the potential health risks and burning conditions of cleaner cooking methods.

Monitoring the user’s adherence to improved stoves represents a challenge and is affected by cultural practices that might have influenced the effectiveness of using ICS in improving respiratory health. Ensuring consistent use of the cookstoves among participants is important, as non-compliance can decrease the intervention’s measurable impact. Due to the restrictions derived from the COVID-19 pandemic, the period between the first assessment and the follow-up had to be prolonged, obstructing the monitoring of the adherence of communities to the ICS. The authors worked to overcome this issue by involving cooperative leaders and performing visits and calls to households between the two assessments. Additionally, we acknowledge the limitations of self-reported symptoms, which are subject to recall bias, reporting bias, and individual variability in symptom perception. To mitigate these potential biases, a standardized and piloted questionnaire, including control questions, was administered by trained interviewers. Lastly, a fraction of the participants dropped out of the study before the second assessment, resulting in a reduction of the sample size by almost 10%. Nevertheless, the remaining participants represented a balanced mixture of individuals using traditional cooking and ICS, enabling cross-sectional and longitudinal comparisons.

### Conclusions

The findings of this study underscore the critical need for interventions that address HAP to improve respiratory health in rural East African communities. While the findings of this study may not be universally applicable to all rural settlements, they provide valuable insights and are particularly representative of sub-Saharan African contexts. Implementing ICS should be regarded as a transitional approach rather than a conclusive solution for cleaner cooking. Programs to reduce HAP may benefit from complementary interventions, such as improving home ventilation, promoting cleaner fuels and energy sources (e.g., solar, LPG, or biogas), and educating communities on safe cooking practices. However, we highlight that under adequate utilization, ICS contribute to alleviating household pollution and could reduce the burden of respiratory diseases as well as the time allocated for cooking, representing benefits for households in terms of productivity and resource utilization.

Further research should focus on several key areas to build upon the findings of this study. First, long-term studies are necessary to assess the sustained health impacts of ICS use, particularly on respiratory and cardiovascular health, over extended periods. Additionally, further studies should incorporate comprehensive air quality measurements, including UFP, gaseous, and emerging pollutants, to better understand the pollution profile associated with ICS use and its long-term health effects. Special attention must be given to sensitive populations, such as pregnant women, children, and older adults. By addressing these gaps, future research will help refine strategies for mitigating household air pollution and improving public health in resource-limited regions.

## Supplementary Information

Below is the link to the electronic supplementary material.

Supplementary material 1 (PDF 692.4 kb)

## Data Availability

Access to the datasets created in this study is available from the corresponding authors upon reasonable request. Participants’ clinical information will only be provided, grouped, and anonymized.
