# Supplementary material for: Improved cookstoves enhance household air quality and respiratory health in rural Rwanda
Source: Sci Rep. 2025 Jul 18;15:26065. doi: 10.1038/s41598-025-09863-6 (PMC12274399; doi:10.1038/s41598-025-09863-6)
Supplement: Supplementary file 1 — Supplementary material 1 (PDF 692.4 kb) [file 41598_2025_9863_MOESM1_ESM.pdf]

Supplementary Information of the Manuscript:

Improved cookstoves enhance household air quality and respiratory health in rural Rwanda

Andrea Cuesta-Mosquera, Henning Kothe, Leizel Madueno, Allan Mubiru, Christine Muhongerva, Thomas Müller, Jan Rupp, Dominik van Pinxteren, Manuela van Pinxteren, Katherine Ogurtsova, Vanessa Soppa, Miriam Wiese-Posselt, Mira Pöhlker

Study sites

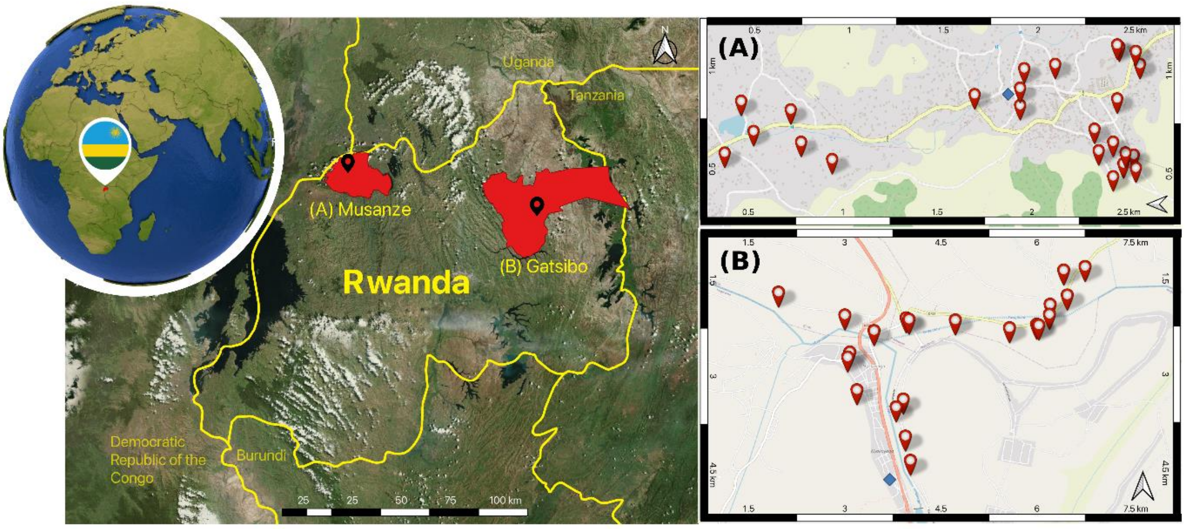

Figure S1: Measurement locations for household air pollution in Musanze (A) and Gatsibo (B), Rwanda. Red pins indicate household approximate locations where measurements were conducted, and the blue diamond marks the site of the first and second phases of health assessments. The map on the left shows the two districts (Musanze and Gatsibo) within Rwanda, with national boundaries and neighboring countries.

Other configurations of the traditional cooking and the heat-retaining box, Wonderbox

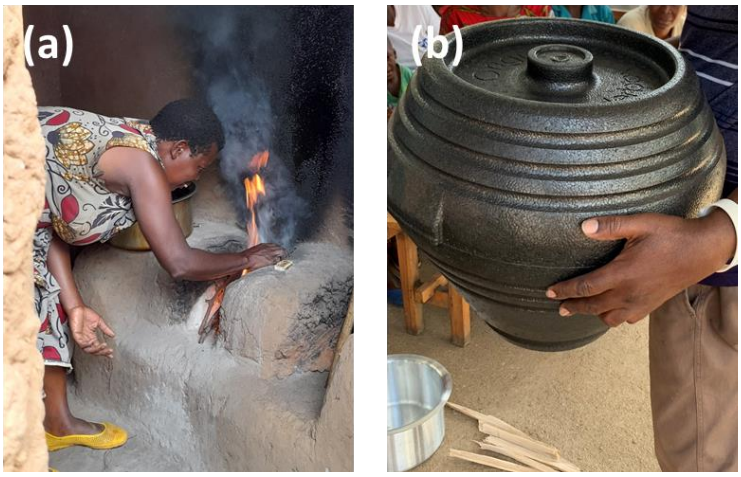

Figure S1: (a.) U-shaped traditional cooking setting. (b.) Heat retaining box (Wonderbox®).

Considerations to recalculate BC mass concentrations from MA200

BC mass concentrations were recalculated to correct for the loading effect (loading compensation) using as an approximation the algorithm proposed by Virkkula et al. for Aethalometers[1]. In summary, BC is corrected as follows:

$$BC_{corrected} = (1 + k * ATN)BC_0 \qquad \text{(equation S1)},$$

where k is an empirical constant (also named compensation factor), ATN is the attenuation coefficient, and BC_0 is the noncorrected BC concentration given by the instrument. The compensation factor k is calculated as follows:

$$k \approx \frac{1}{ATN(t_{i,last})} * \left(\frac{BC_0(ATN(t_{i+1,first}))}{BC_0(ATN(t_{i,last})} - 1\right) \qquad \text{(equation S2)},$$

Where $t_{i,last}$ is the time of the last measurement done on the spot i and $t_{i+1,first}$ is the first measurement for the next (new) spot i+1. We have determined values of k for several households covering all wavelengths monitored by the MA200 and for each cooking method. We estimated and used median values of k, which are shown below.

Table S1: MA200 compensation factors estimated from field measurements.

**Wavelength (nm)**	***k***	
	**Traditional cooking**	**Improved cookstove**
375	0.023	-0.003
470	0.016	-0.005
528	0.015	-0.007
625	0.013	-0.009
880	0.009	-0.015

Stages during online HAP measurements (example for one household)

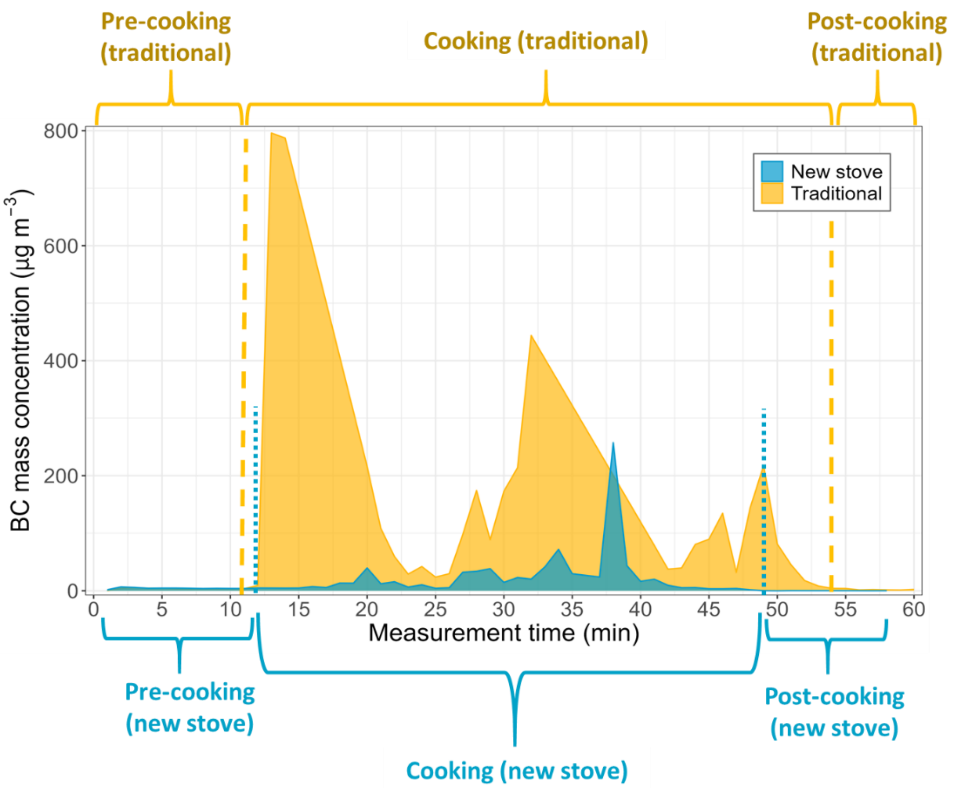

Figure S3: Example of time-resolved measurements of BC mass concentrations during traditional cooking and ICS use before (*pre-cooking*), during (*cooking*), and after (*post-*) cooking.

Calculation of PM concentrations from OPSS data

PM concentrations were calculated online using OPSS aerosol size distribution measurements (300-1000 nm). To calculate PM mass concentrations from the PNSD, we assumed spherical aerosol particles with a density of 1.4 g cm^{-3} [2]. The mass size distribution was calculated as follows:

$$\frac{dM}{dlogDp} = \frac{dN}{dlogDp} * \left(\frac{Dp}{1000}\right)^3 * \frac{\pi}{6} * \rho,$$

where Dp is the particle size bin, ρ is the particle density, and $\frac{dN}{dlogDp}$ correspond to the particle number concentration.

Particle size accuracy tests and calibration in the OPS were performed using polystyrene latex (PSL) particles of known sizes. We applied a refractive index correction to adjust the scattering-based measurements of the aerosols monitored indoors since their optical properties differ from those of PSL. We used a complex refractive index representative of biomass-burning aerosol particles[3].

OPS flow calibration was also performed using a reference mass flowmeter (TSI, model 4040). Deviations in flow after calibration were <10%.

Quality assurance and quality control of online measuring instruments

Before the field measurements, the optical instruments were calibrated in the laboratory and compared against reference instruments at the World Calibration Centre for Aerosol Physics (WCCAP) in Leipzig, Germany. Additionally, quality checks were performed every day before measurements. BC mass concentrations measured by the MA200s were compared to a Multiangle Absorption Photometer (MAAP, Thermo, model 5012) measurements. The MAAP determines BC mass concentrations based on measurements of aerosol light attenuation at 637 nm[4]. The instrument is a light absorption photometer that collects aerosol particles over a filter material and uses the light absorption properties of black carbon (BC) to determine its concentration. An internal algorithm converts the BC absorption into BC mass concentration using a mass absorption cross-section of 6.6 $m^2 g^{-1}$. We used ambient air for the comparison. The comparison to the reference showed deviations in a range of 10-20%. The deviations found from the comparison with MAAP were in the range of values reported in the literature[5]. The unit-to-unit comparison resulted in a ~10% unit-to-unit variability (see figure below).

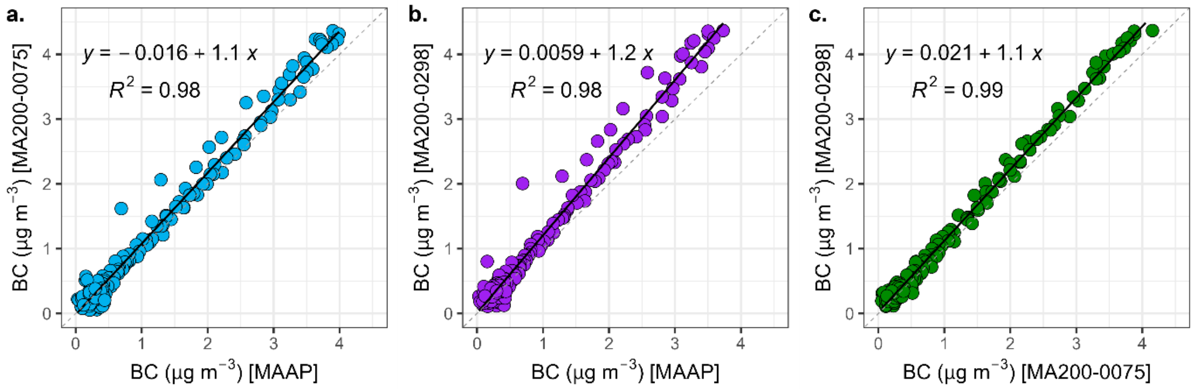

Figure S4: Scatterplots from the intercomparison of the portable absorption photometers (MA200) against a reference MAAP (a. and b.) and the MA200 unit-to-unit comparison (c.). The figures include the regression equations and coefficients of determination (R^2). The black solid lines correspond to the linear model fit, and the gray dashed lines represent 1:1 lines.

Background BrC exposure measured before cooking

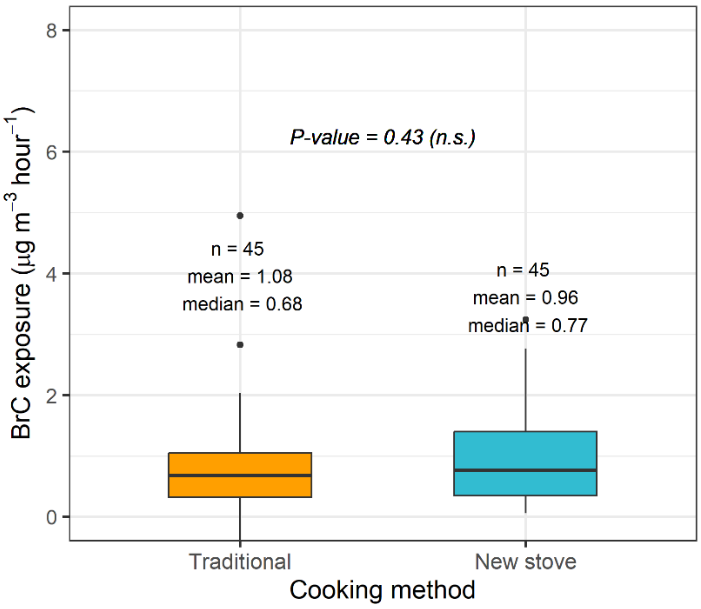

Figure S4: Boxplots of pre-cooking BrC exposure measured for both cooking methods during HAP monitoring. The lower and upper borders of the boxes represent the first and third quartiles in which the middle 50% of the statistical variables are located, the horizontal black lines inside the boxes represent the median, and the whiskers represent the minimum and maximum values without outliers. The black dots represent the outliers.

References

1. Virkkula, A. *et al.* A simple procedure for correcting loading effects of aethalometer data. *J Air Waste Manage Assoc* **57**, 1214–1222 (2007).
2. Turpin, B. J. & Lim, H. J. Species contributions to pm2.5 mass concentrations: Revisiting common assumptions for estimating organic mass. *Aerosol Science and Technology* **35**, 602–610 (2001).
3. Kim, J. *et al.* Assessing optical properties and refractive index of combustion aerosol particles through combined experimental and modeling studies. *Aerosol Science and Technology* **49**, 340–350 (2015).
4. Müller, T. *et al.* Characterization and intercomparison of aerosol absorption photometers: Result of two intercomparison workshops. *Atmos Meas Tech* **4**, 245–268 (2011).
5. Alas, H. D. C. *et al.* Performance of microaethalometers: Real-world field intercomparisons from multiple mobile measurement campaigns in different atmospheric environments. *Aerosol Air Qual Res* **20**, 2640–2653 (2020).
